# A Half-Century History of Applications of Antisense Oligonucleotides in Medicine, Agriculture and Forestry: We Should Continue the Journey

**DOI:** 10.3390/molecules23061302

**Published:** 2018-05-29

**Authors:** Volodymyr V. Oberemok, Kateryna V. Laikova, Anna I. Repetskaya, Igor M. Kenyo, Mikhail V. Gorlov, Igor N. Kasich, Alisa M. Krasnodubets, Nikita V. Gal’chinsky, Iryna I. Fomochkina, Aleksei S. Zaitsev, Viktoriya V. Bekirova, Eleonora E. Seidosmanova, Ksenia I. Dydik, Anna O. Meshcheryakova, Sergey A. Nazarov, Natalya N. Smagliy, Edie L. Chelengerova, Alina A. Kulanova, Karim Deri, Mikhail V. Subbotkin, Refat Z. Useinov, Maksym N. Shumskykh, Anatoly V. Kubyshkin

**Affiliations:** 1 Taurida Academy, V.I. Vernadsky Crimean Federal University, Vernadsky Avenue 4, 295007 Simferopol, Crimea; tn-nov@mail.ru (A.M.K.); pcr.product@gmail.com (N.V.G.); zaycevfl@mail.ru(A.S.Z.); viktoriya.bekirova@ya.ru (V.V.B.); elya.seidosmanova@mail.ru (E.E.S.); fenol.ksu@gmail.com (K.I.D.); list10@mail.ru (A.O.M.); puplivebros@yandex.ua (S.A.N.); scarletsun7991@mail.ru (N.N.S.); tchelengerova@yandex.ru(E.L.C.); UseinovRefat@gmail.com (R.Z.U.); 2 Medical Academy named after S.I. Georgievsky, V.I. Vernadsky Crimean Federal University, Lenin Avenue 5/7, 295051 Simferopol, Crimea; botan_icus@mail.ru (K.V.L.); fomochkina_i@mail.ru (I.I.F.); kulanovalina@gmail.com (A.A.K.); deri97@mail.ru (K.D.); msgerm@yandex.ru (M.V.S.); kubyshkin_av@mail.ru (A.V.K.); 3 Botanical Garden named after N.V. Bagrov, V.I. Vernadsky Crimean Federal University, Vernadsky Avenue 4, 29500 Simferopol, Crimea; anna.repetskaya@gmail.com; 4 Academy of Bioresources and Environmental Management of V.I. Vernadsky Crimean Federal University, 95492 Agrarnoye, Crimea; kenyo_i_m@mail.ru; 5 D. Mendeleev University of Chemical Technology of Russia, Miusskaya sq. 9, 125047 Moscow, Russia; mikgorlov@gmail.com; 6 Rostov State Medical University, Nakhchivan Lane 29, 344022 Rostov-on-Don, Russia; igrkas@gmail.com

**Keywords:** antisense oligonucleotides, antisense therapy, DNA insecticides, RNAi, medicine, agriculture, forestry

## Abstract

Antisense oligonucleotides (ASO), short single-stranded polymers based on DNA or RNA chemistries and synthesized in vitro, regulate gene expression by binding in a sequence-specific manner to an RNA target. The functional activity and selectivity in the action of ASOs largely depends on the combination of nitrogenous bases in a target sequence. This simple and natural property of nucleic acids provides an attractive route by which scientists can create different ASO-based techniques. Over the last 50 years, planned and realized applications in the field of antisense and nucleic acid nanotechnologies have produced astonishing results and posed new challenges for further developments, exemplifying the essence of the post-genomic era. Today the majority of ASOs are chemically modified and/or incorporated within nanoparticles to enhance their stability and cellular uptake. This review critically analyzes some successful cases using the antisense approach in medicine to address severe diseases, such as Duchenne muscular dystrophy and spinal muscular atrophy, and suggests some prospective directions for future research. We also examine in detail the elaboration of unmodified insect-specific DNA insecticides and RNA preparations in the areas of agriculture and forestry, a relatively new branch of ASO that allows circumvention of the use of non-selective chemical insecticides. When considering the variety of successful ASO modifications with an efficient signal-to-noise ratio of action, coupled with the affordability of in vitro oligonucleotide synthesis and post-synthesis procedures, we predict that the next half-century will produce a fruitful yield of tools created from effective ASO-based end products.

## 1. Introduction

Although the history of science is broad and complex, we feel confident providing an analysis of important research, facts, and findings in this particular field of interest: nucleic acid-based gene silencing/modification. The literature on ASOs is extensive and we apologize for having to be selective. Practical application of the principles of antisense oligonucleotides (ASOs) was first formulated in Novosibirsk (Russia) by Grineva in 1967 [1]. She proposed that attachment of active chemical groups to oligonucleotides directs these groups to a certain fragment of a nucleic acid complementary to the oligonucleotide, so that the chemical reaction (modification) occurs in a specific region of the target nucleic acid near the formed duplex structure. This approach was called a “method of complementary-addressed modification”. In 1977, using this antisense approach to modify valine tRNA, Grineva and colleagues demonstrated that the method allows alkylation with a reagent bound to the corresponding oligonucleotide at certain points along the valine tRNA [2].

In 1972, the Nobel laureate Gobind Khorana created a strategy, using overlapping DNA oligonucleotides, for synthesis of a DNA duplex with a sequence corresponding to the major yeast alanine transfer RNA [3]. Two years later, Letsinger and coworkers introduced the phosphite coupling approach, which was a key innovation in oligonucleotide synthesis using phosphoramidates [4]. This approach has been adapted to the synthesis of deoxyoligonucleotides [5], oligoribonucleotides [6], and nucleic acid analogs [7]. In 1977, Paterson and colleagues were the first to publish research showing that gene expression can be modified with exogenous nucleic acids by use of single-stranded DNA to inhibit translation of complementary RNA in a cell-free system [8].

In 1978, Zamecnik and Stephenson used a specific 13-nt-long oligodeoxynucleotide to inhibit Rous sarcoma virus replication and cell transformation in chicken embryo [9]. In the same set of experiments, the authors improved the activity of the oligonucleotide by introducing chemical modifications at the 3′ and 5′ ends, which reduced its degradation by cellular nucleases. This successful attempt using ASO as a molecular tool for in vivo cell management is considered the first such attempt documented in the literature. One year later, in 1979, Donis-Keller described results showing that RNase H cleaves the RNA strand in RNA-DNA heteroduplexes in a site-specific manner [10], providing evidence that antisense oligonucleotides operate via an enzyme-mediated process to degrade target RNA. The 1970s can be viewed as the decade that gave birth to the antisense approach. The findings presented in the research described above demonstrated that antisense oligonucleotides, which inhibit gene expression in a sequence specific way, could be used to create practical solutions to real problems.

Unfortunately, until the mid-1980s, further progress stalled, primarily for three reasons. First, it was believed that an oligonucleotide’s hydrophilic sugar-phosphate backbone prevented it from entering eukaryotic cells. Second, synthesis of a target oligonucleotide was difficult and time consuming; the procedures and equipment needed to automate oligonucleotide (ON) synthesis had not yet been developed [11,12]. Third, genomic sequence information for humans and other target organisms was scarce [13]. In 1983, evidence for the existence of naturally occurring antisense RNAs, and their role in the regulation of gene expression, was first published [14]. In a breakthrough with respect to use in eukaryotic cells, the technique successfully inhibited both the translation of exogenous RNA injected into *Xenopus laevis* oocytes [15,16], and the translation of endogenous mRNA [17]. The importance of these results lies in their clear demonstration of the idea that antisense oligonucleotides could be used to manipulate gene expression in living cells [18].

Until the late 90s, translation of that idea into practical applications for use in medicine was more dream than reality, and its existence was absolutely unknown to researchers in agriculture and forestry. Fomivirsen, the first antisense drug made with a phosphorothioate backbone, was approved in 1998 by the U.S. Food and Drug Administration (U.S.FDA) and in 1999 by the European Agency for the Evaluation of Medicinal Products (EMEA) for the treatment of retinitis cytomegalovirus (CMV) in patients with AIDS. Although fomivirsen was administered locally as an intravitreal injection, its use demonstrated the possibility that drugs developed from antisense oligonucleotides could be administered systemically in the treatment of human diseases [19]. The target of fomivirsen was the mRNA that encoded the CMV immediate-early (IE)-2 protein, which is required for viral replication. At the time, there was an urgent unmet need for an anti-cytomegalovirus retinitis drug. Subsequently, due to the development of high-activity anti-retroviral therapy (HAART), the number of CMV cases has dramatically decreased. Novartis stopped marketing fomivirsen in Europe in 2002 and in the United States in 2006 [20].

Also in 1998, Fire and colleagues showed that site-specific double-stranded RNA is responsible for downregulation of target genes in the nematode *Caenorhabditis elegans* [21]. It has been hypothesized that this results from a simple antisense mechanism dependent on hybridization between the injected RNA and endogenous messenger RNA transcripts, because the later steps of the RNA interference were carried out by antisense RNA molecules, analogs of DNA ASO. Of note, in animals, the first clues about RNAi were unearthed during the use of sense and antisense RNA oligonucleotides by Guo and Kemphues [22] in a series of experiments conducted with RNA antisense in the nematode *C. elegans*. As expected, analysis of the expression of the par-1 gene showed that it was silenced when nematodes were treated with RNA antisense for par-1. However, in control experiments using RNA sense, par-1 expression was also impaired. The above-referenced work of Fire and colleagues, who carefully purified the RNA antisense, RNA sense, and double-stranded RNA (dsRNA) for the gene unc-22 of *C. elegans*, provided an explanation for this paradoxical result. The results of experiments examining unc-22 interference showed that single-stranded RNAs (either sense or antisense) were 10 to 100 times less effective than dsRNA. It was found that single-stranded sense RNAs were effective only if delivered immediately before or after the antisense RNA. This suggests that the sense and antisense strands hybridized in vivo to form a dsRNA, which was then responsible for the activity seen. The notion that RNAi could be used to develop a new class of therapeutics fired the imagination of many investigators soon after its discovery, providing the impetus needed to move the field of applied RNAi therapeutics swiftly from lab to bedside [23]. The RNAi approach has been applied to the development of new drugs and several phases II and III clinical trials currently being carried out [24]. However, there are still some concerns and challenges to be overcome as therapeutic applications are being developed and tested. These include, but are not limited to, the possibility of causing off-target effects, triggering innate immune responses and, both important and complex, determining the best way to deliver the drug into the cytoplasm of target cells.

Later, in the first few years of the current century, a few scientists have envisaged the use of RNA interference (RNAi) itself as an insect pest control tool by targeting vital genes [25], including those of transgenic plants engineered to express dsRNAs, which would be ingested by the target insects [26,27]. Using RNAi and double-stranded RNA (dsRNA) fragments to create non-chemical pesticides has potential in the ongoing effort to control insect pests, particularly lepidopterans [28,29]. However, this approach has several drawbacks. It is possible that the efficacy of RNAi action in lepidopteran insects could be compromised because: (i) some insect genes are resistant to RNAi [30]; (ii) RNA substrate synthesis is both costly and time consuming; and (iii) delivering the product to the targeted insects under field conditions is challenging [29]. In addition, the selectivity of this approach is significantly hampered because the relatively long dsRNAs in cells are cleaved into numerous, very short siRNAs (21–23 nucleotides in length) with abundant direct sequence matches among the genomes of non-target organisms [31].

In 2008, we proposed for the first time the use of topical application of nucleic acids in the control of phytophagous insects. We created short, unmodified antisense DNA insecticide for use against the gypsy moth *Lymantria dispar*, a major insect pest of hardwood trees; application of these insecticides was followed by significant larval mortality [32,33]. Three years later, Wang and colleagues created dsRNA fragments specific for the Asian corn borer *Ostrinia furnacalis*; topical application was followed by significant larval mortality in this case as well [34]. As oral administration was originally believed to be the only possible way to deliver double-stranded RNAs to target tissues, other than injection, because the insect midgut is not protected by chitin integument [29], our results and those of Wang et al. ably demonstrated that topical application was a viable option. Topical application of single-stranded DNA or double-stranded RNA may induce mortality by passing into interior tissues via the tracheal system, which is not covered by chitinous exoskeleton [28], or via diffusion through the soft thin cuticle of young instars. Several factors make unmodified nucleic acids strong candidates for use as insecticides: selectivity, fast biodegradation compared with conventional chemical insecticides, and more affordable commercial synthesis of nucleic acids in vitro.

**Figure 1:**
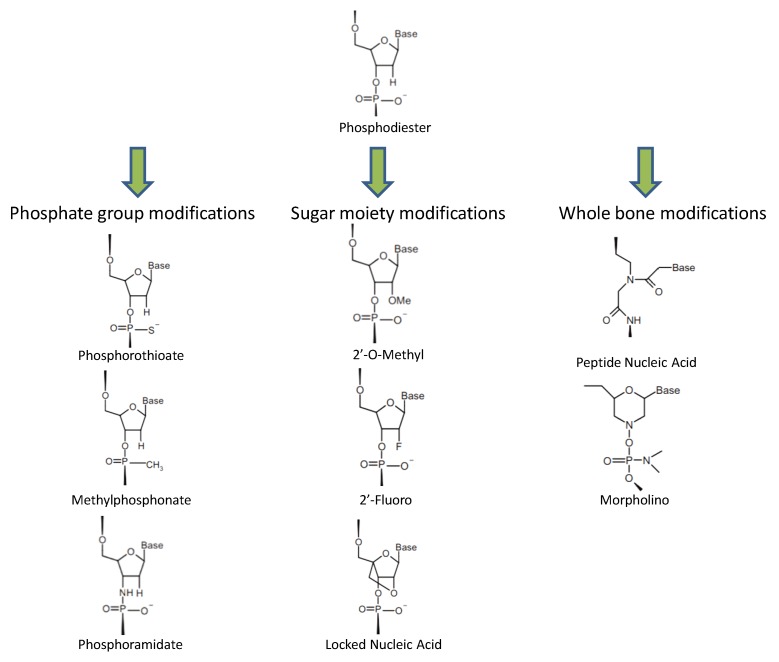
The main chemically modified antisense oligonucleotides.

While promising, there are caveats to the use of unmodified antisense oligonucleotides as a tool to develop insecticides. An important feature that allows the creation of unmodified DNA insecticides is the weaker activity of insect enzymes (e.g., esterases) compared with those of mammals [35]. Phosphodiester oligonucleotides are limited for use medically in mammalian tissues because they are rapidly degraded by intracellular endonucleases and exonucleases [36]. These degradation products, deoxynucleotide 5′-monophoshate (dNMP) 2-mononucleotides, may be cytotoxic and could exert antiproliferative effects [37]. For instance, Koziolkiewicz and colleagues [38] have shown a relationship between that the toxic effects of dNMPs and mononucleotide dephosphorylation by the cell-surface enzyme ecto-5′-nucleotidase. Scientists have developed many chemical modifications of ASOs in an attempt to overcome the side effects connected with application of unmodified nucleic acids and expand the arsenal made possible by this approach (Figure 1). In a moment, we will discuss the story of three remarkable generations of ASO modifications. Before we begin, however, it is important to keep in mind that all antisense techniques, with the possible exception of those employed by the animal itself and/or recruited unmodified nucleic acids, are subject to off-target effects.

## 2. ASO Diversity and Their Functional Activities

Phosphorothioate (PS) ASOs, which contain a non-bridging sulfur, are the most widely studied oligonucleotides; compared to unmodified oligonucleotides, they possess higher solubility and nuclease stability, with improved membrane penetration (Table 1). The study of phosphorothioate biochemistry began with the synthesis of adenosine 5′-phophorothioate, which was unexpectedly resistant to the phosphatases that normally degrade adenosine 5′-phosphate [39]. Therapeutic use of oligonucleotides is highly dependent upon their efficient uptake by cells. The literature contains many reports of experiments both in tissue culture and in vivo using this backbone to generate antisense effects mediated by a RNase H-dependent mechanism. These data have led to the investigation of phosphorothioate oligonucleotides in therapeutic clinical trials [36]. However, when sulfur replaces one of the nonbridging oxygens at each phosphorus in the oligonucleotide chain, chirality is introduced. In fact, only the Sp phosphorothioate diastereomer is nuclease resistant; the Rp diastereomer is nuclease sensitive. However, the Sp linkage is sterically helix destabilizing, which decreases the melting temperature (Tm) of the oligonucleotide/mRNA complexes relative to the natural phosphodiester oligomer [40]. Few of these first generation ASOs reached late-stage clinical trials, and only fomivirsen (Vitravene1) was approved and marketed for the intraocular treatment of cytomegalovirus retinitis [36]. The interactions between PS-ASOs and other cellular proteins have not been well characterized. In addition, these molecules have been shown to induce sequence independent, but length dependent, binding to various cellular proteins, especially heparin-binding molecules, such as laminin and fibronectin [41].

The RNase H-dependent phosphorothioate oligonucleotides produce a vast array of nonspecific effects. Efforts to increase specificity have inspired researchers to mix and match oligonucleotide chemistries [36], leading to the development of second-generation ASOs with additional modifications to their sugar backbone moieties. These modifications confer much greater nuclease resistance and increased binding affinities (the latter often related to the reduced flexibility of nucleoside rings) compared with those of earlier PSs or phosphodiesters [42]. The drugs developed from second-generation RNase H-dependent ASOs (~20 nucleotides long) are constructed as ‘gapmer’, with 10 deoxynucleotides flanked at either end by five ribonucleotides (5-10-5 gapmer). Additionally, these ASOs have a PS backbone, and the flanking ribonucleotides are modified with 2′-O-methyloxyethyl, to improve stability and potency. These PS-ASOs base-pair with the target RNAs in the cell and trigger sequence-specific cleavage of the RNA by RNase H1, which is present in both cytoplasm and nucleus [43].

Third generation ASOs include a wide variety of molecules: we would like to highlight three of them, namely, morpholino oligomers (MOs), peptide nucleic acids (PNAs), and locked nucleic acids (LNAs). Several representatives from among the abovementioned ASOs (particularly the LNAs) are currently under investigation in clinical trials being carried out by a number of biotechnology companies [44]. MOs, synthetic oligonucleotides comprising chains ~25 subunits in length, are similar to DNA and RNA oligonucleotides, with substitution of a morpholine ring for the ribose ring found in DNA and RNA. While MOs still undergo Watson-Crick base pairing, this substitution confers significant advantages to MOs compared with conventional oligonucleotides [45]. Morpholino oligomers should have far fewer nonspecific properties than phosphorothioate oligos, since they are not charged; as a result, they should also be less toxic. MOs are remarkably stable because they are resistant to nucleases. Since the effects of MOs are not mediated via an RNase H mechanism, they can be designed to inhibit translation [46] and to interfere with the proper splicing of RNA employing a steric block mechanism [47]. Morpholino oligomers bind RNA with a higher affinity than DNA, and a much higher affinity than PS ASOs [48] (Table 1). Unsurprisingly, like other ASOs, the main problem with MOs is that they can actually produce off-target effects. For example, MOs may prevent correct splicing and form a completely irrelevant gene product. Some MOs may activate p53 and consequent p53-induced apoptosis. The mechanism by which some MOs activate p53 is unknown for the moment. The literature contains two clear examples of research that demonstrates these off-target effects, work carried out in sea urchins and zebrafish [49]. Unfortunately, it is highly likely that there are other cases, many as yet unrecognized, where non-specific phenotypes caused by MOs have occurred. When assessing the probabilities in a pragmatic fashion, the more pessimistic calculations suggest that, for every two MOs introduced into a developing embryo, one will elicit nonspecific effects.

Peptide nucleic acids (PNAs), like MOs, are oligonucleotides that carry out their primary functions using a simple steric block mechanism. PNAs have a charge-free polyamide scaffold of repeating *N*-(2-aminoethyl) glycine units, with the nucleobases attached via methylene carbonyl linkers, in place of the negatively charged ribose-phosphate backbone found in other oligonucleotides [36]. This change allows them to hybridize with either DNA or RNA in a sequence specific manner. PNAs are primarily used in situations in which very high binding affinities were required. The absence of negative charges on the PNA oligomers, with its resulting lack of electrostatic repulsion, provides an explanation for the high-affinity nucleic acid binding observed. Unfortunately, poor cellular penetration and inadequate in vivo pharmacokinetic properties have thwarted efforts to use PNAs therapeutically [50] and they have fallen out of favor as a treatment modality. However, since PNAs are easy to synthesize and make functional, and are both more stable and more responsive to point-mutations than their DNA counterparts, fluorogenic PNAs represent an interesting alternative to DNA-based molecular beacons for use in sensing applications in a cell-free environment, where cellular uptake is not required [51].

In locked nucleic acid (LNA)-modified oligonucleotides, a number of natural nucleotides has been replaced with nucleotide analogs that feature an altered sugar moiety, with the ribose 2′-O- and 4′-C-atoms connected via a methylene bridge [52]. The effects of LNAs are mediated primarily through RNase H [53]. Owing to their potentially lower immunostimulatory potential (as compared with PS ASOs), along with relatively high potency, short sequences, and favorable binding affinities, trials incorporating LNA backbones are currently being carried out by several different companies interested in developing ASO therapeutics [44,54]. Structurally, LNA oligonucleotides are conformationally rigid. Each LNA residue introduced into a duplex comprising DNA or RNA strands increases its melting temperature by several degrees, which, along with the rigid conformation of the LNA residues, contributes to the stability of the duplex [55,56]. In the presence of transfection reagents in cell culture, LNA oligonucleotides have low single-digit nanomolar or high picomolar IC50 values for mRNA down-modulation [57,58]. In general, LNAs (i) lack the unspecific toxicity of phosphorothioates; (ii) have potential side targets with much lower accessibility than that of the intentional target; and (iii) do not create major functional consequences with most side targets, if accessible [52]. Essentially, all aspects of antisense technology have benefitted from work done with LNAs because of their unusually high affinity for target sequence, greatly improved mismatch discrimination, general low toxicity, and increased metabolic stability [52]. However, they do appear to have a higher potential for hepatotoxicity and some other specific toxic effects [59,60].

An additional class of nucleic acid therapies exists whose pathophysiologic effects are mediated via a completely different mechanism, in which double-stranded RNA can be used to silence specific genes [44] and thus the resulting protein expression. The therapeutic potential for drugs developed using RNAi is wide open, as proteins represent the largest group of pharmacological drug targets [24]. Delivery of RNAi based drugs to the target cells and tissues remains a major challenge that has hampered antisense DNA oligonucleotide technologies from the beginning. The need for safe and effective in vivo RNAi drug applications makes it imperative that delivery of double-stranded small interfering (siRNAs) to specific cells be addressed. siRNAs are small and carry a negative charge, increasing the difficulty they encounter when attempting to cross the cell membrane. A variety of delivery strategies exist, which include, but are not limited to: nanoparticles, cationic lipids, antibodies, cholesterol, aptamers, and viral vectors for short hairpin RNAs [23]. Core components of the siRNA-mediated post-transcriptional silencing (PTGS) include the RNase III enzyme Dicer and its co-factor transactivating response RNA-binding protein, along with the Argonaute family of proteins. Two distinct mechanisms regulate PTGS: translational repression and degradation of mRNAs with imperfect complementarity, and sequence-specific cleavage of perfectly complementary mRNAs. Exogenous siRNAs, or short hairpin RNAs (shRNAs) that share perfect or near-perfect Watson-Crick base pairing with the intended mRNA target, are able to exploit the sequence-specific cleavage mechanism [61]. siRNA created when Dicer converts dsRNAs into duplexes 21–25 nt long, with 3′ 2 nt overhangs, is incorporated into one or more of the Argonaute proteins in the RNA induced silencing complex (RISC) [62], which functions in the cytosol rather than in the nucleus. By unwinding the two-stranded RNA molecules, RISC liberates the antisense strand, allowing it to bind to the targeted RNA molecule, where endonuclease activity hydrolyzes the site where the antisense strand is bound [63]. While most current antisense therapeutics have been developed to target mRNA, one clinical drug uses antisense technology to inhibit an endogenous microRNA (miRNA) [61]. Endogenous 18–25 nt long miRNAs induce translational repression and mRNA degradation when the guide (antisense) strand has limited complementarity with the target mRNA. The dysregulation of endogenous miRNAs has been linked to numerous diseases, including many types of cancer [64]. The toxicologic profiles of siRNA differ from those of the more traditional ASOs; for this reason, a safety concern unique to therapeutic siRNAs is the unintended suppression of non-target mRNAs and proteins via RNA interference (RNAi). While their effects are mediated via different mechanisms, both ASOs and siRNAs can induce hybridization-dependent off-target effects [23]. Current Phase II and Phase III clinical trials are underway for nine small-interfering RNAs (siRNAs), as well as one unique microRNA (miRNA) inhibitor, all of which are racing to the pharmaceutical market [24].

## 3. Four Recent Successful Applications of ASO to Medicine

**Figure 2:**
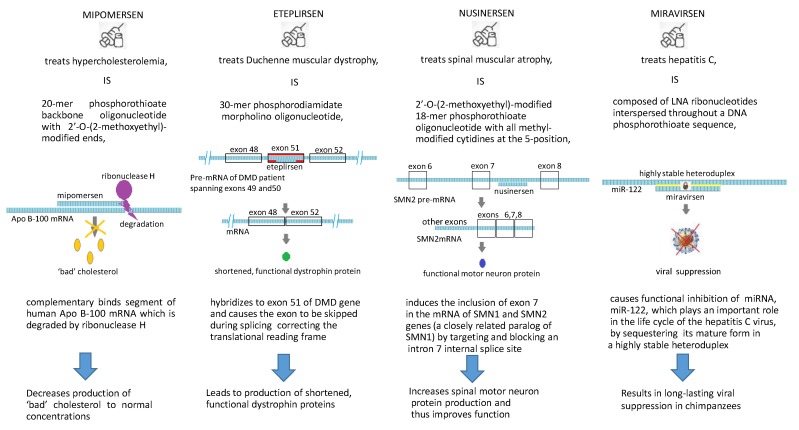
The scheme of action of mipomirsen, nusinersen, eteplirsen, and miravirsen.

With respect to the era of clinical trials, in this section we will briefly introduce four successful drugs with different ASO chemistries and treatment targets that have reached, or almost reached, the manufacture stage (Figure 2). Various drug delivery systems have been explored to improve the bioavailability of nucleic acids, and nanoparticles (NPs) have been suggested as potential vectors for DNA/RNA. Although the current applications of ASOs to therapeutic medicine seem exotic, since they aim to treat quite rare diseases, it is fitting to pay tribute to these drugs and their developers, because they chose to address difficult conditions, with seemingly unreachable goals, where conventional approaches had not noticeably succeeded.

### 3.1. Mipomersen (Kynamro^®^)

Kynamro^®^ (mipomersen, Genzyme/Isis) was approved by the U.S. FDA in January 2013 for use in the treatment of familial hypercholesterolemia (FH) [65,66]. FH, the most common inherited cause of premature coronary heart disease, entails significant morbidity and mortality [27], with a prevalence of approximately 1 person in every 200–500 among those who are heterozygotes in North America and Europe [67]. The drug, developed as a 20-mer of nucleotides (polynucleotide of 20 bases), has a sequence complementary to that of a segment of human Apo B-100 mRNA. It has a phosphorothioate backbone, with 2′-*O*-(2-methoxyethyl)-modified ends, which when compared with earlier antisense technologies, provides greater biological stability and a higher binding affinity to the target mRNA [68].

Antisense drugs in general, and mipomersen in particular, are routed to the liver following injection; the liver is also the site of Apo B-100 synthesis. Reduction of Apo B-100 synthesis in the liver decreases production of low-density lipoprotein and very-low-density lipoprotein particles (often referred to as ‘bad’ cholesterol) to normal concentrations [69]. Tissue endonucleases metabolize mipomersen, forming shorter oligonucleotides, which are further metabolized by exonucleases [65]. Clearance of mipomersen from body tissues was found to be slow in all species studied. It begins with the nuclease metabolism in the tissues, followed by predominantly urinary excretion of both the parent drug and its chain-shortened metabolites [70]. The most frequently observed on-treatment adverse events were mild-to moderate injection-site reactions and flu-like symptoms [66]. In general, the results achieved with mipomersen point to its efficacy, safety, and tolerability, demonstrating its suitability for use in the target patient population, and providing a tangible tool for use in the management of FH and severe hypercholesterolemia.

### 3.2. Nusinersen (Spinraza^®^)

Spinraza^®^ (Biogne), approved by the U.S. FDA in December 2016, is used in the treatment of spinal muscular atrophy (SMA) [20]. SMA, a leading genetic cause of pediatric mortality, is an autosomal recessive neuromuscular disease caused by progressive loss of α-motor neurons in the anterior horn of the spinal cord [71]. Based on the age of onset and subsequent clinical course, three different forms of childhood SMA are currently recognized: Werdnig-Hoffmann disease (SMA1), the intermediate form (SMA2), and Kugelberg-Welander disease (SMA3) [72]. In all cases, SMA results from a mutation of the survival motor neuron 1 (SMN1) gene on chromosome 5, leading to a structural/functional deficiency of the survival motor neuron (SMN) protein. Nusinersen, an 18-mer phosphorothioate 2′-O-methoxyethoxy antisense oligonucleotide, has had all of its cytidines methyl-modified at the 5-position. The oligo induces the inclusion of exon 7 in the mRNA of two genes (SMN1 and SMN2 (a closely related paralog of SMN1)) [73] by targeting and blocking an intron 7 internal splice site [20]. These actions increase SMN protein production and thus improve function [74]. Intrathecal injection of nusinersen allows therapeutic delivery directly into the cerebrospinal fluid bathing the spinal cord, the site of motor neuron degeneration. In one of the studies, 89% of participants overall reported adverse events (AE). The AEs most frequently reported by study participants following treatment with nusinersen were headache (39.3%), post-lumbar puncture headache (21.4%), and back pain (17.9%). While the most common side effects of nusinersen are respiratory infections and constipation, the FDA has also issued warnings with respect to the possibility of thrombocytopenia and renal toxicity [75,76].

### 3.3. Eteplirsen (EXONDYS 51^TM^)

Eteplirsen (EXONDYS 51^TM^, Sarepta) was approved by the USFDA in September 2016, making it the first and currently only FDA-approved drug for Duchenne muscular dystrophy (DMD); additionally, it is the first morpholino drug ever approved. DMD is a progressive, disabling genetic neuromuscular disorder, occurring only in males, caused by an absence of dystrophin, with an incidence of 1 in 3500–5000 males born worldwide [77,78]. This absence is the result of mutations in a gene coding for dystrophin [79,80], a membrane-associated protein that forms a network with sarcolemmal glycoproteins by linking the cytoskeletal actin in muscle fibers with the surrounding extracellular matrix [81]. In children affected by DMD, the disorder begins within the first few months of life, causing the development of muscle weakness. It progresses rapidly, so that they lose the ability to walk during childhood. This universally fatal disease culminates in early death, often when these young men are still in their 20s, most commonly as the result of respiratory or cardiac complications [79].

As is the case with other morpholino oligomers, eteplirsen avoids the unwanted degradation of possibly important nontarget transcripts by not employing the RNase H system. By hybridizing to exon 51 of DMD gene, the drug causes the exon to be skipped during splicing [82,83], correcting the translational reading frame. This leads to production of shortened, functional dystrophin proteins [79].Eteplirsen, a phosphorodiamidate morpholino oligomer 30 nucleotides in length [79], with the sequence CTCCAACATCAAGGAAGATGGCATTTCT [82], is administered via intravenous infusion and was found to be well tolerated, with no adverse effects, in several clinical trials [79].

### 3.4. Miravirsen (SPC3649)

Miravirsen (SPC3649, Santaris Pharma), an anti-MiR drug candidate that has entered Phase II clinical trials [84] for treatment of HCV infections [85], comprises LNA ribonucleotides interspersed throughout a DNA phosphorothioate sequence complementary to that of mature miR-122. These LNA modifications increase both the drug’s resistance to nuclease degradation and its affinity for its target, MiR-122, a liver-specific miRNA that plays an important role in the life cycle of the hepatitis C virus (HCV). Most people infected with HCV develop chronic hepatitis C viral infections, which increase their risk of cirrhosis and can lead to serious clinical complications such as hepatocellular carcinoma [86,87]. Miravirsen causes functional inhibition of miR-122 by sequestering mature miR-122 in a highly stable heteroduplex [88]. In a study done using an animal model, administration of miravirsen to chimpanzees with chronic HCV infections resulted in long-lasting viral suppression; there was no evidence of any resistant mutations at either of the two miR-122 binding sites of the 5′ untranslated region of the HCV genome [89]. In a Phase I study in healthy human volunteers, no adverse events were observed [90]. Phase II clinical trials of Miravirsen carried out in patients with chronic HCV demonstrated a significant, dose-dependent decrease in HCV, which proved to be sustainable for some time after administration. In general, the synthetic RNAi-based drugs that survived development and are currently involved in Phase II and Phase III clinical trials (bevasiranib, patisiran, QPI-1002, etc.) have shown that they are able to silence a wide variety of genes. These genes are found in diseases whose deleterious effects manifest in distinctly different ways, including cell death caused by apoptosis or by uncontrolled cell proliferation. Clinical trials have shown that these products are safe and well tolerated. Most of the adverse effects observed were rated as mild to moderate, occurring primarily during either local or systemic drug injection. Infusion-related reactions that occurred following intravenous injection of the drug were generally transient and could be prevented by pretreatment with glucocorticoids, antihistamines, or other anti-inflammatory agents [24].

## 4. ASO-Based Insect Pest Management in Agriculture and Forestry

Despite the fact that every year new insecticides are developed for plant protection, the cost of losses caused by insect pests remains at the same levels, about 30% pre-crop losses and 10% post-crop [91]. Taking into consideration the rapid population growth worldwide, the annual reduction of cultivated areas, and substantial losses from insect pests, most experts believe that no serious alternative to chemical insecticides exists because they help to preserve 20% of all crops [92]. Due to the inevitable genetic resistance emerging over time for every pesticide preparation [93,94], the development, production, and implementation of new insecticides is a never-ending task in agriculture and forestry.

Currently, there are three distinct groups of insecticides: chemical (neonicotinoids, pyrethroids, carbamates, etc.), biological (bacterial, viral, and fungal), and those occupying an intermediate position between chemical and biological, DNA insecticides and RNA preparations. Chemical insecticides are affordable and fast acting, but they have long half-lives (often of environmental concern) and are non-selective. On the other hand, while biological preparations are selective, they are slow acting and relatively expensive to produce. DNA insecticides and RNA preparations are inherently chemical insecticides made of natural polymers that, due to the principle of complementarity, act in a highly selective manner. Thus, DNA insecticides and RNA preparations are able to unite the best characteristics of the other types of modern insecticides, without the disadvantages, and can be synthesized in huge quantities on automatic equipment. Below we will discuss the main advantages and disadvantages of nucleic acid-based preparations and the prospects for their creation. Although research using an ASO-based approach is still in its infancy in the areas of agriculture and forestry, its application is no less promising than it is in medicine. Although the use of phosphodiester oligonucleotides to develop drugs for use in mammalian tissues is limited, their use in developing insecticides is extremely promising. Since the activity of insect enzymes (e.g., esterases) is weaker than those of mammals [35], the use of this approach to discover and develop insecticides is not hampered by the rapid degradation of oligonucleotides by intracellular endonucleases and exonucleases observed in mammals [36]. This paves the way for the prospective use of unmodified antisense oligonucleotides as a tool for insect pest control.

### 4.1. RNA Preparations

While reluctance to publish negative results may have led some researchers to overestimate the general susceptibility of insects to RNAi techniques [25], research efforts focusing on exploring the potential of insecticides developed using RNA interference (RNAi) to suppress crop pests have made huge strides [95]. Nevertheless, in this section, we will outline in detail the shortcomings of RNA interference to better understand the most obvious problems encountered when creating these preparations.

Interest in developing RNAi products for insect pest control has intensified in recent years, not only for use in a more traditional manner, applied as an insecticide, but also as structures occurring within genetically modified (GM) plants [68]. As with many other applications of genetic engineering in plants, the use of RNAi to develop new traits in plants has sparked a debate about the environmental safety of its use, and was the subject of a symposium session at the 13th ISBGMO in Cape Town, South Africa in 2014 [96]. Along with using RNAi to develop therapies for human diseases, one of the earliest non-research applications envisaged was the use of plant expressed dsRNA to confer resistance to insects; the first reports of its effectiveness were in 2007 [26,97].

RNAi activity is based on sequence homology between small RNAs and mRNAs. An unintended consequence of this, and thus a challenge in the development of useful approaches, is that off-target genes with sufficient sequence homology to the siRNA generated may also be silenced. Thus, even though the target genes were silenced, the therapies have the potential to cause adverse effects to the health of animals and humans, as well as to the environment. Since plants are also living organisms, the off-target gene silencing could occur within the GM plant itself, or in other organisms that are exposed to or ingest the GM plant [68,98]. Scientists have thus come to the conclusion that use of this technology, particularly in the case of GM plants, should be accompanied by rigorous environmental risk assessments that take into consideration the potential for harm to non-target organisms. Clearly, our current understanding of how environmental exposure to dsRNA affects plants, animals, and insects, as well as which parameters are most likely to be responsible for off-target gene effects, is far from complete [96]. While bioinformatic analyses could, in the future, play an important role in assessing the risk of RNAi-based GM plants, at present, they are unreliable for use alone, without other methods, to predict the presence of RNAi activity. More research is needed to determine the exact rules that govern small RNA-target matches; armed with this knowledge, researchers can design more efficient algorithms, leading to more reliable predictions. In addition, it is absolutely necessary for us to expand our knowledge of animal and insect genomes and their expression, especially in little-studied lines and in a variety of species [99]. Several agricultural companies are currently working on better methods to produce ready-to-spray RNAi products at a low cost. This represents a more practical and timely approach compared with the creation of genetically modified organisms, which is costly in both production and time, and subject to regulatory hurdles from governmental agencies and outcry and resistance from the public [55].

The spectrum of sensitivity of arthropods to ingested dsRNA varies widely [25]; among the various orders of arthropod, coleopterans demonstrate a significantly higher sensitivity. Compared with that of the coleopterans, the susceptibility to ingested dsRNA among lepidopteran species varies considerably and requires higher concentrations of dsRNA to elicit a response [30]. Insect response when exposed to environmental dsRNA also varies greatly, as does the ability to elicit systemic RNAi, whether it is administered via microinjection or by other means [25,30,100]. Once it has entered an insect’s system, the dsRNA (generally fewer than 1000 nt long) is cleaved into much smaller siRNAs (consistently ~21–23 nt long), which may be amplified intracellularly [101]. It is worth noting that evidence of this amplification has rarely been observed in either insect (a primary target of RNAi-based GM crops) or mammals [102,103]. Research into developing useful insecticides based on dsRNA explores the hypothesis that uptake of dsRNA from the environment via ingestion will lead to transport of the RNAi signal to the cells and other body tissues. To date, a precise understanding of this process and how it functions in insects remains elusive, which continues to hamper several potential practical applications that would be useful in controlling insect pests. From a pest control perspective, the absence of a functional systemic RNAi system results in ineffective knockdown or a knockdown with merely a localized effect (i.e., only in the midgut, where dsRNA uptake occurs), which may or may not cause mortality [55].

RNAi efficiency in insects is subject to myriad competing factors. These include the concentration of dsRNA, the lengths of the various dsRNA fragments, the timing and duration of exposure, the extent of both dsRNA uptake and degradation, activation of the RNAi machinery, and the life stage of the target organisms [30,51,104,105]. To achieve high levels of silencing, researchers in most studies have injected a ‘standard’ amount of dsRNA, ranging between 1 and 100 µg. Routine injection with amounts of dsRNA that fall within this standard range, regardless of the size of the targeted species, has led to calculated sensitivities to RNAi (the amount of dsRNA administered per mg tissue required to achieve silencing) that appear to be higher in larger species [30]. Determining the best length and optimal concentration of exogenous dsRNA is absolutely necessary when working to create effective RNAi. This is made more challenging by the fact that the length of dsRNA required to elicit an effective RNAi response varies depending upon insect species [7,30]. Most studies in the literature have reported that dsRNA ranging from 140 to 500 nucleotides in length are required to achieve successful RNAi [55]. While the production of relatively long dsRNA for research purposes can be accomplished using commercially available molecular biology kits, the price per microgram of such in vitro synthesized dsRNA is too high to be commercially viable [55]. To address the need for large amounts of dsRNA, development of more cost-efficient mass production methods is currently underway, including bacterial production and synthetic nucleoside triphosphate (NTP) modifications [106].

**Figure 3:**
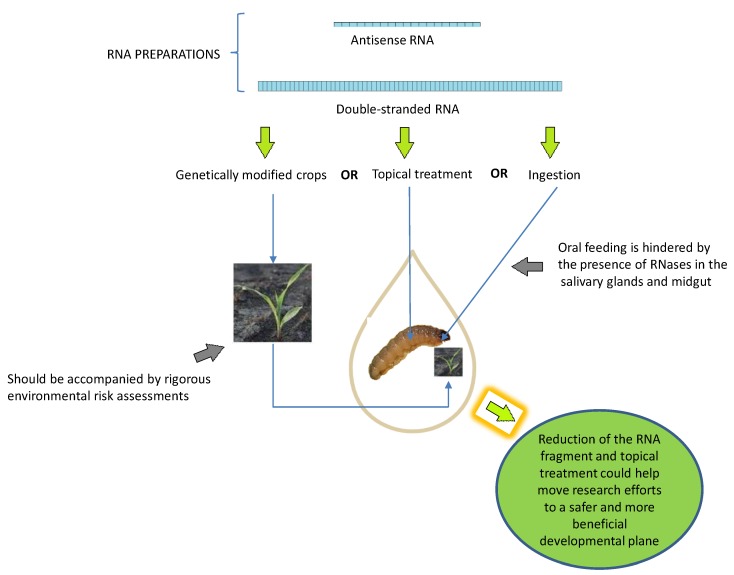
Perspectives of RNA preparations in agriculture.

The three most common methods used to deliver RNAi are microinjection, ingestion, and soaking. Microinjection is extremely useful for introducing accurate amounts of dsRNA exactly into the chosen area. Insect ingestion of dsRNA is the most useful method for large-scale gene screening and is the main source of publications regarding RNAi control of insect pests. For example, administering double-stranded RNA by feeding, as seen in *Epyphia spostvittana* [107] and *Rhodnius prolixus* [108], paves the way in this field. Soaking is convenient for research on insect cells [109]. Oral feeding is hindered by the presence of dsRNases in the salivary glands and midgut of many insects, which makes the pests recalcitrant to RNAi [55]; microinjection, while useful in the laboratory, is not a feasible method of insect pest control in a field or forest. Among the listed delivery methods, topical treatment seems to be the most promising, since the majority of successful conventional chemical insecticides belong to the category of contact insecticides. Despite the obvious barriers, it has been demonstrated that uptake of dsRNA by the entire insect body is possible. Wang and colleagues sprayed dsRNA directly on newly hatched *Ostrinia furnacalis* larvae [34]. This treatment resulted in extensive mortality, which ranged from 40% to 100%, and correlated with the downregulation of the target gene expression as verified by qPCR. This easily applied delivery method clearly demonstrated that it is possible for dsRNAs to penetrate the insect integument and trigger RNAi, providing concrete evidence that RNAi-based pest control can be facilitated by use of this high-throughput dsRNA delivery method. Unfortunately, the number of publications available concerning the contact use of RNA preparations is limited, since research into using this application is not widespread. Apparently, the large relative length of the dsRNA fragments used plays a critical role in this matter. In our opinion, reduction of the length of the RNA fragment needed, coupled with evidence supporting its use as a contact application, could help move research efforts for RNA preparations to a safer and more beneficial developmental plane (Figure 3), very close to that of DNA insecticides.

### 4.2. DNA Insecticides

For the last 10 years, our group has conducted work on creation of DNA insecticides for the gypsy moth *Lymantria dispar* L. control [32,33,110,111,112]. We chose the gypsy moth as the target insect for our investigations because gypsy moth larvae, who cause damage to over 500 plant species, are therefore directly responsible for substantial economic losses. Gypsy moth larvae are voracious feeders, able to consume more than 1 m^2^ of foliage per larva during the caterpillar stage [113,114]. During surges in population (outbreaks), which can last 1–3 years, larvae are capable of completely defoliating host trees, after which they move on to cereal crops and even vegetables. The population densities of the gypsy moth (*Lymantria dispar*; Lepidoptera: Lymantriidae) are able to reach outbreak levels that pose considerable economic losses, to forests in Europe, Asia, Africa, North America [115], and even New Zealand [116].

Data from our most recent laboratory research demonstrate that unmodified antisense DNA oligonucleotides from the RING (really interesting new gene) domain of the LdMNPV (*Lymantria dispar* multiple nucleopolyhedrovirus) IAP-3 (inhibitor-of-apoptosis) gene have pronounced insecticidal effects on LdMNPV-free [32,33,112] and LdMNPV-infected gypsy moth larvae [110]. The results obtained suggest that in LdMNPV-free and LdMNPV-infected gypsy moth larvae, the oligoRING (our main DNA insecticide; 5′-CGA CGT GGT GGC ACG GCG-3′) acts as an antisense RNase H-dependent oligonucleotide inducing the degradation of target mRNA for the LdMNPV IAP-3 and host IAP-Z (very homologous to LdMNPV IAP-3) genes, followed by subsequent downregulation of target protein expression. Results obtained for insect mortality, biomass accumulation, histological studies, and analysis of DNA apoptotic fragmentation suggest that, while the oligoRING induces apoptotic processes in both LdMNPV-free and LdMNPV-infected insect cells, they were more pronounced in the latter. Of note, some LdMNPV-free larvae reared by us from egg masses under laboratory conditions did not show any significant sensitivity to the oligoRING fragment, which intensifies our interest in the deeper investigation of the phenomenon of DNA insecticides.

Among natural populations of the insect, we observed strong insecticidal and metabolic effects as the result of treatment with a single-stranded antisense DNA fragment from the RING domain of the gypsy moth LdMNPV IAP-3 gene: specifically, reduction of biomass (by 35%) and decreased survival of *L. dispar* larvae. Treatment with this DNA fragment led to significantly lower survival rates among female insects (1.7 fold), accompanied by signs of apoptosis. Additionally, we found calcium and magnesium imbalances in the eggs laid by the surviving females treated with the RING DNA fragment, indicating that the strong stress reactions and metabolic effects were not confined only to the treated insects, but are likely to have led to apoptosis in the eggs as well. This is important, because one of the most pressing challenges when creating DNA insecticides is the need to increase insect mortality, which at the moment is no more than 40–50%, compared to the mortality seen in control, which generally does not exceed 10%. Unfortunately, to increase the effectiveness, DNA insecticide formulation may require addition of carriers, which will make this approach less affordable.

This proposed new approach for insect pest management, an advance in the field of ‘microbial pesticides’, is based on application of the specific virus DNA, created as the result of the knowledge gained about virus-pest interactions, being put to use for the benefit of mankind. It is noteworthy that the consequences of just one treatment with the viral antisense DNA fragment were observed during the entire life cycle of the treated insects as well as in the next generation. This suggests that the viral DNA is able to coordinate particular cellular pathways, both as a part of a whole viral genome and also as short fragments of it. This field of research is of fundamental importance, especially in light of the surge of interest in the interaction between host and DNA viruses in humans. Based on observations of the significant role played by ssDNA, we suppose the general existence of mechanisms of eukaryotic cell regulation carried out by short ssDNA fragments of DNA-containing viruses during their degradation by intracellular nucleases, which require further investigation [33].

One such observation is that baculovirus IAPs bear a striking resemblance to the cellular IAPs carried by the host insects that they infect [117]. Cellular IAPs, members of a highly conserved family of survival factors, are responsible for the regulation of developmental- and stress-induced apoptosis, as well as inflammation, the cell cycle, and some signaling processes [118]. For this reason, homologous IAP found in *Lymantria dispar* (our investigations show it to be host IAP-Z gene homologous to LdMNPV IAP-3 gene) provides an ideal target for apoptosis induction (most of the baculovirus IAPs with anti-apoptotic functions belong to the IAP-3 group, with certain exceptions [119]) following application of oligoDNA and the subsequent death of the whole insect. Although many insect pest genomes have not yet been sequenced, a considerable number of insect virus genomes are already known. The virus genes of cellular origin will be invaluable in development of plant protection based on nucleic acids in the near future, because they contain combinations of nitrogenous bases that have shown their effectiveness in the host-virus relationship system for millions of years. This is important because, by allowing researchers to target genes for pest management that are already tied to a specific pair virus-host (for example, anti-apoptosis genes), it reduces the likelihood that target genes will be silenced in non-target organisms and helps lower possible environmental risks. Data from our recent studies show that DNA insecticides designed for gypsy moth larvae can be selective, and thus non-harmful both for non-target insects, such as black cutworm and tobacco hornworm [111,112], and for plants such as wheat [120], oaks, and apple trees [121]. Positive results like these open the door to the creation of selective insecticides that are well-tailored to target pest insects, while sparing other insects and plants [112].

There are a number of reasons why DNA insecticides are more advantageous than elaborate RNA preparations against lepidopterans at the larval stage. First, short (~18–20 nt long) insect-specific DNA insecticides are more affordable to produce compared to relatively long double-stranded RNA fragments. Second, high concentrations of double-stranded RNA are required to achieve successful gene silencing if the RNA is administered by feeding or injection. Both of these issues could be resolved by using topical application of DNA insecticides based on short antisense DNA fragments that produce results at substantially lower concentrations. In most studies, the ‘standard’ amount of double-stranded RNA injected to achieve high levels of RNAi (but not death) in insects varies between 1 and 100 μg [30]. In experiments with DNA insecticides, in contrast, topical application of 3–30 pmol viral DNA fragments (18 nt long) per gypsy moth larva led to significant mortality. This range corresponds to approximately 1.8–180 ng DNA per mg of larval biomass. Therefore, ssDNA-based insecticides function well at substantially lower concentrations; accordingly, insecticides developed using this approach may be more affordable than RNA preparations for insect pest control. In some cases, such as in attempts to control secretive insects and adult beetles, it may be impossible to use DNA insecticides, because elytra may provide some protection from contact insecticides. Nevertheless, DNA insecticides appear to be excellent candidates for insect pest control of non-secretive lepidopteran pests at the larval stage, especially during early larval instars, when the insects’ exoskeletons are thin. Third, very short 18 nt long pest-specific antisense DNA fragments (DNA insecticide) will not be cleaved in the cells of non-target organisms, unlike relatively long dsRNAs, which are diced into very short siRNAs that may silence non-target genes [68]. Last, the presence of the 2′-OH group makes the hydrolysis of RNA much more facile than hydrolysis of DNA [122]. Thus, DNA insecticides will be more stable than RNA preparations under natural conditions, allowing them to exert a greater insecticidal effect before being degraded. Finally, at the moment, there is not a single publication to be found detailing research or industry that uses the RNAi approach for gypsy moth control.

**Figure 4:**
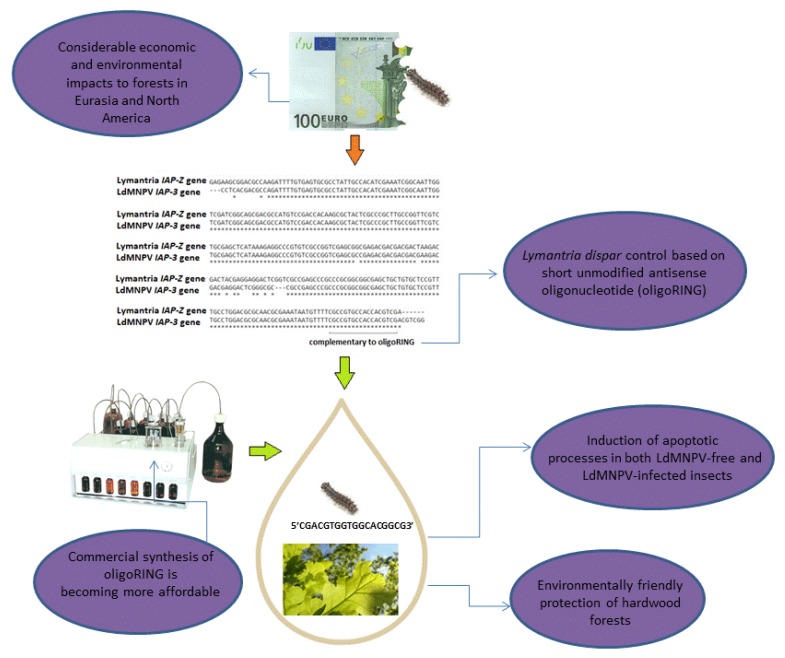
Perspectives of DNA insecticides for gypsy moth control.

The RING domain is a highly conserved segment of insect and baculovirus IAP genes, which makes it an excellent research target. Searches of the GenBank NIH genetic sequence database for the conserved antisense segment inside the RING domain fragment in the LdMNPV IAP-3 gene revealed the 5′-CGA CGT GGT GGC ACG GCG-3′ sequence. This fragment, which is found in all LdMNPV genomes represented in GenBank, is considered to be well conserved, and we decided to use it as oligoDNA (oligoRING) for the treatments. In our opinion, use of DNA insecticides could resolve, or at least improve upon, the important problem of insecticide resistance by slowing it down. The use of short single-stranded fragments of highly conserved segments of insect host anti-apoptosis genes should slow development of resistance to these insecticides, because research has shown that the potential mutations responsible for changes to the target anti-apoptosis genes occur at a very low rate in the conserved parts of the genes. Thus, even if we cannot halt the genetic processes leading to insecticide resistance, we may be able to slow down its emergence using DNA insecticides based on very conservative regions of functionally important genes, such as anti-apoptosis genes. This innovative approach is immensely valuable, even revolutionary, and further work in this field may lead to economical, environmentally safe agriculture enabled and sustained by DNA insecticides. Our calculations suggest that today the cost to treat 1 hectare of oak forest in Russia and Ukraine with DNA insecticides is comparable to the cost to treat the same area of forest with a chemical preparation, Dimilin^®^ (diflubenzuron). This chemical is a gypsy moth suppressant [123] that acts by non-selective inhibition of the biosynthesis of arthropod chitin [124]. This suggests that the use of DNA insecticides is economically justified and just around the corner (Figure 4).

## 5. Experiments and Ready-to-Use Preparations Have always Been Stronger than Words of Skepticism

There is no doubt that in the past 50 years antisense technologies have been responsible for tremendous advances in medicine, agriculture, and forestry. Some of these achievements are not yet visible to non-professionals. The greatest and at the same time potentially most limited results have been achieved in the area of medicine. To the credit of the antisense approach, it is difficult to imagine what other approaches could successfully offer drugs for the treatment of Duchenne muscular dystrophy or spinal muscular atrophy. After spinning outward from Watson and Crick’s double helix DNA structure of 1953, we have spiraled back, finding ways to use nucleic acids as a universal tool for cell management, although in almost all cases modified by additional groups. Undoubtedly, while the drugs appearing on the market represent great progress, these potentially life-saving substances also carry extremely high price tags attached by their developers. There is a hope that some pushback will emerge from insurance carriers or governmental authorities to mitigate this. In general, it is expected that the next few decades will see the appearance of a large number of ASO-based drugs. Several impediments to their widespread use as drugs still have to be overcome. The most serious of these are their lack of stability in physiological fluids and their poor penetration into cells. It is already impossible to stop the avalanche, since the idea of using antisense oligonucleotides is too elegant to go unnoticed, much less forgotten. Of course, in some cases, this approach cannot be successfully applied, especially in emergency situations. In our opinion, various approaches are ideally suited for various diseases; in the case of antisense oligonucleotides, they are well suited to use in developing treatments for genetic disorders and chronic viral infections requiring long-term use of drugs with minimal side effects. 

Agriculture and forestry have lagged behind medicine in researching the practical applications of the antisense approach. It should be noted that, unlike medicine, in agriculture and forestry, there exists a competition between DNA insecticides and RNA preparations based on unmodified oligonucleotides. Insect pest control always involves getting a significant portion of the preparation used into the environment, which frequently comprises an area much larger than one human patient. Unmodified oligonucleotides seem to be the safest way to do this, since cells contain ubiquitous nucleases that can neutralize them. The longest lasting insecticidal effect occurs only in the case of an insect pest with the mRNA of the target gene. In our opinion, RNA preparations rank behind DNA insecticides in almost all respects, including affordability and selectivity in action, but they are winning in popularity. Today’s attitude towards DNA insecticides resembles that of the 70s and 80s in the 20th century, when the majority of the scientific community was skeptical of the possible applications of unmodified antisense oligodeoxyribonucleotides to problems in medicine, let alone those in agriculture and forestry. However, insect enzymes (including nucleases) are weaker than those of mammals, paving the way for the creation DNA insecticides and RNA preparations. In any case, experiments and ready-to-use preparations are always stronger than words of skepticism. Despite the fact that it is much easier to obtain a license for a preparation destined for use in agriculture and forestry than for one used in medicine, no end-products yet exist, but, undoubtedly, they will soon appear. After all, change in medicine has always occurred at a more intensive rate than in agriculture and forestry. We should be patient and continue the journey.

## Figures and Tables

**Table 1:** Summary of properties of three generations of antisense oligonucleotides in comparison with phosphodiesters.

ASOs	Ability to Penetrate Cells	Stability in Vivo	Binding to Target mRNA	Level of Efficiency	Non-Target Effects
Phosphophodiesters (antisense RNA and DNA, dsRNA)	Moderate	Moderate	Moderate	Moderate	Moderate
First generation (phosphorothioates, methylphosphonates)	Moderate	Higher nuclease stability	Lower binding capacity	Higher effectiveness	More pronounced non-specific effects
Second generation (2′-*O*-methyl and 2′-*O*-methyloxyethyl oligonucleotides)	Moderate	Much higher nuclease stability	Higher binding capacity	Higher effectiveness	More pronounced non-specific effects
Third generation (locked nucleic acids, peptide nucleic acids, morpholino oligomers)	Higher penetration ability	Nuclease resistant	Much higher binding capacity	Much higher effectiveness	Moderate
